# Emergency Minimally Invasive Esophagogastrectomy and Delayed Esophagocoloplasty for Caustic Ingestion: A Stepwise Video-Based Approach

**DOI:** 10.7759/cureus.114384

**Published:** 2026-08-11

**Authors:** Gianluca Marcocci, Sara Roveta, Massimo Ciciliot, Angelo Franceschi, Raffaele Galleano

**Affiliations:** 1 General and Oncologic Surgery, Ospedale San Paolo, Savona, ITA

**Keywords:** caustic ingestion, colon interposition, coloplasty, esophagectomy, esophagocolic anastomosis, feeding jejunostomy, gastrectomy, mediastinoscopy, minimally invasive surgery, transhiatal esophagectomy

## Abstract

Caustic ingestion is a rare but life-threatening condition that may require emergency surgery. While open surgery remains the standard approach, minimally invasive strategies are increasingly explored. Given the rarity of these cases, a standardized and reproducible approach aimed at improving outcomes is needed. In this context, a standardized stepwise technique for emergency minimally invasive total gastrectomy and transhiatal subtotal esophagectomy with cervical esophagostomy, followed by delayed reconstruction using transverse colon interposition, is described. Indications strictly followed World Society of Emergency Surgery (WSES) guidelines (Zargar ≥3a, transmural necrosis, or perforation). Six consecutive patients underwent emergency surgery. Mean operative time was 242 minutes, with one postoperative death due to sepsis. Delayed reconstruction was performed in four patients after a median of 9.5 months, with satisfactory clinical outcomes and no anastomotic leaks. Description of key technical steps is supported by a full-length annotated surgical video. A minimally invasive transhiatal approach is feasible in selected patients and may facilitate delayed reconstruction. Standardization of technique is essential to improve reproducibility in this challenging setting.

## Introduction

Caustic ingestion is a life-threatening condition requiring prompt multidisciplinary management. The extent of injury depends primarily on the type and quantity of the ingested agent and represents the main determinant of prognosis and therapeutic strategy.

Conservative or surgical management is determined by the combined assessment of clinical, endoscopic, and radiological findings. Upper gastrointestinal endoscopy, which should ideally be performed within three to 48 hours after caustic ingestion, allows accurate evaluation of the extent and severity of endoluminal mucosal injury.

The Zargar classification is the most widely adopted endoscopic grading system and includes the following categories: Grade 0: normal mucosa; Grade 1: mucosal edema and hyperemia; Grade 2a: superficial, localized, non-circumferential ulcerations with mucosal friability and blister formation; Grade 2b: deep, circumferential ulcerations; Grade 3a: multiple deep ulcerations with scattered, limited areas of focal necrosis; Grade 3b: extensive transmural necrosis; and Grade 4: perforation.

However, endoscopy cannot accurately predict the depth and transmural extent of tissue necrosis. Then, contrast-enhanced computed tomography (CT) is routinely integrated into the diagnostic workup to identify transmural injury and help therapeutic decision-making. A radiological classification of esophagogastric caustic injuries distinguishes three grades: Grade 1: normal-appearing wall; Grade 2: wall edema with inflammatory changes in the surrounding soft tissues and increased post-contrast mural enhancement; and Grade 3: transmural necrosis, characterized by the absence of post-contrast mural enhancement.

The combined assessment of endoscopic and radiological findings stratifies caustic injuries into low-grade and high-grade lesions. Low-grade injuries (Zargar Grades 0-2a and radiological Grade 1) can be safely managed conservatively, whereas high-grade injuries (Zargar Grades ≥2b and radiological Grades 2-3) require close clinical monitoring and may warrant surgical intervention [1].

Overall, epidemiological data are limited and often underestimated: in France, approximately 350 cases occurred annually, of which about 11% require surgical intervention. Patients undergoing emergency surgery after massive ingestion exhibit high morbidity and mortality rates, reported up to 76% and 25%, respectively. Surgical mortality further varies according to the extent of resection, ranging from 17% for isolated esophagectomy or gastrectomy to 37% for multivisceral resections [2].

World Society of Emergency Surgery (WSES) guidelines clearly define indications for emergency surgery in patients with Zargar grade ≥3a injuries, transmural necrosis, or perforation [3,4]. However, the optimal surgical approach remains controversial, particularly regarding the role of minimally invasive techniques in the emergency setting. At present, the most commonly performed surgical approach for esophagogastric emergencies remains laparotomy combined with lateral thoracotomy (usually right, though a left approach is reported) or transhiatal blunt esophageal stripping through a closed chest, the latter being performed without direct visualization of the mediastinum [5-8]. In this context, few authors have advocated a minimally invasive transhiatal approach which enables transabdominal esophageal dissection under magnified visualization while avoiding thoracotomy and its potential respiratory morbidity [7-11].

Given the rarity, high morbidity and mortality, the heterogeneity of surgical strategies described in the literature, and the limited personal experience [1,8], a standardized and reproducible approach aimed at improving outcomes is needed. In this setting, a stepwise minimally invasive transhiatal esophagogastrectomy and conventional cervical coloplasty are described, providing a detailed technical explanation and their preliminary feasibility.

## Technical report

Between June 2021 and March 2026, six consecutive patients (three males, three females; mean age 55 years, median age 53, range 44-80) underwent emergency minimally invasive total gastrectomy, transhiatal subtotal esophagectomy, cervical esophagostomy, and feeding jejunostomy for extensive caustic injuries involving the esophagus and stomach. All patients underwent CT and esophagogastroduodenoscopy (EGD) within 24 hours for Zargar grading. Surgical indication was established in strict accordance with WSES guidelines for radiological evidence of transmural necrosis and/or perforation or Zargar ≥3a. Four of these patients subsequently underwent delayed reconstruction (two males, two females; mean age 55 years, median 49, range 44-80) with transverse colon interposition. The surgical technique for both the emergency and reconstructive phases is described step by step in the section below.

Demolitive phase

Patient characteristics, operative time, and short-term postoperative outcomes are summarized in Table 1. The emergency phase had a mean operative time of 242.5 minutes (median 242.5, range 225-270) and a mean hospital stay of 24 days (median 24, range 18-34). One patient died from sepsis on postoperative day 1. The remaining four patients subsequently underwent delayed reconstruction after a mean interval of 9.5 months (median, 9.5 months; range, 8-11 months). The demolitive phase is described in the step-by-step sections below and is further illustrated in the accompanying surgical video (Video 1).

**Table 1:** Perioperative characteristics and postoperative outcomes of emergency minimally invasive esophagogastrectomy. Clavien-Dindo, Clavien-Dindo classification of surgical complications; POD, postoperative day

Patient	Sex	Age (years)	Operative time (min)	Postoperative complications (Clavien-Dindo)	Length of hospital stay (days)	Interval to reconstruction (months)
1	F	53	255	Death on POD 1 (Grade V)	/	/
2	M	45	235	Anemia requiring blood transfusion (Grade II)	24	10
3	F	54	225	None	20	9
4	M	44	220	Pneumonia (Grade II)	18	11
5	F	80	270	Pleural effusion (Grade IIIa), anemia (Grade II), atrial fibrillation (Grade II)	34	8
6	M	56	250	None	25	Awaiting reconstruction

**Video 1:** Minimally invasive esophagogastrectomy. Demolitive phase Steps 1-6.

1. Trocar Placement and Exploratory Laparoscopy

**Figure 1:**
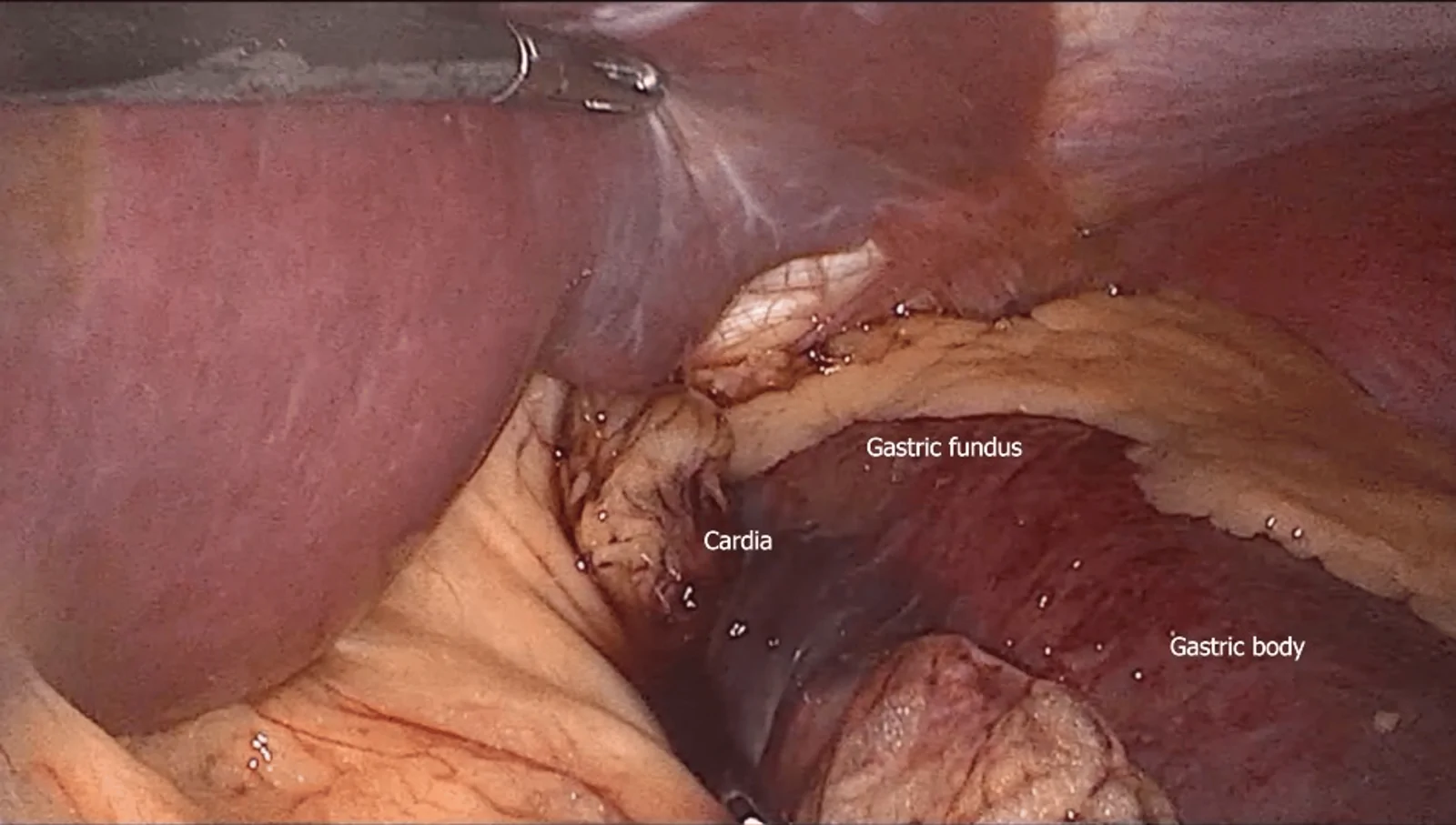
Transmural gastric necrosis. Exploratory laparoscopy to assess the extent of visceral injury and determine the feasibility of a minimally invasive approach.

Patient is supine with legs apart in 15-degree reverse Trendelenburg. Primary surgeon is positioned between legs with an assistant on the right of the patient. Three trocars are placed along a transverse supraumbilical line, and one in the subxiphoid region. The first step is exploratory laparoscopy finalized to quantification of anatomical damage (Figure 1). Conversion to an open approach should be considered in the presence of extensive multivisceral injury (e.g., pancreatic necrosis), diffuse peritonitis, active hemorrhage resulting in massive hemoperitoneum, or persistent hemodynamic instability despite vasopressor therapy.

2. Mobilization of the Greater Curvature and Duodenal Transection

**Figure 2:**
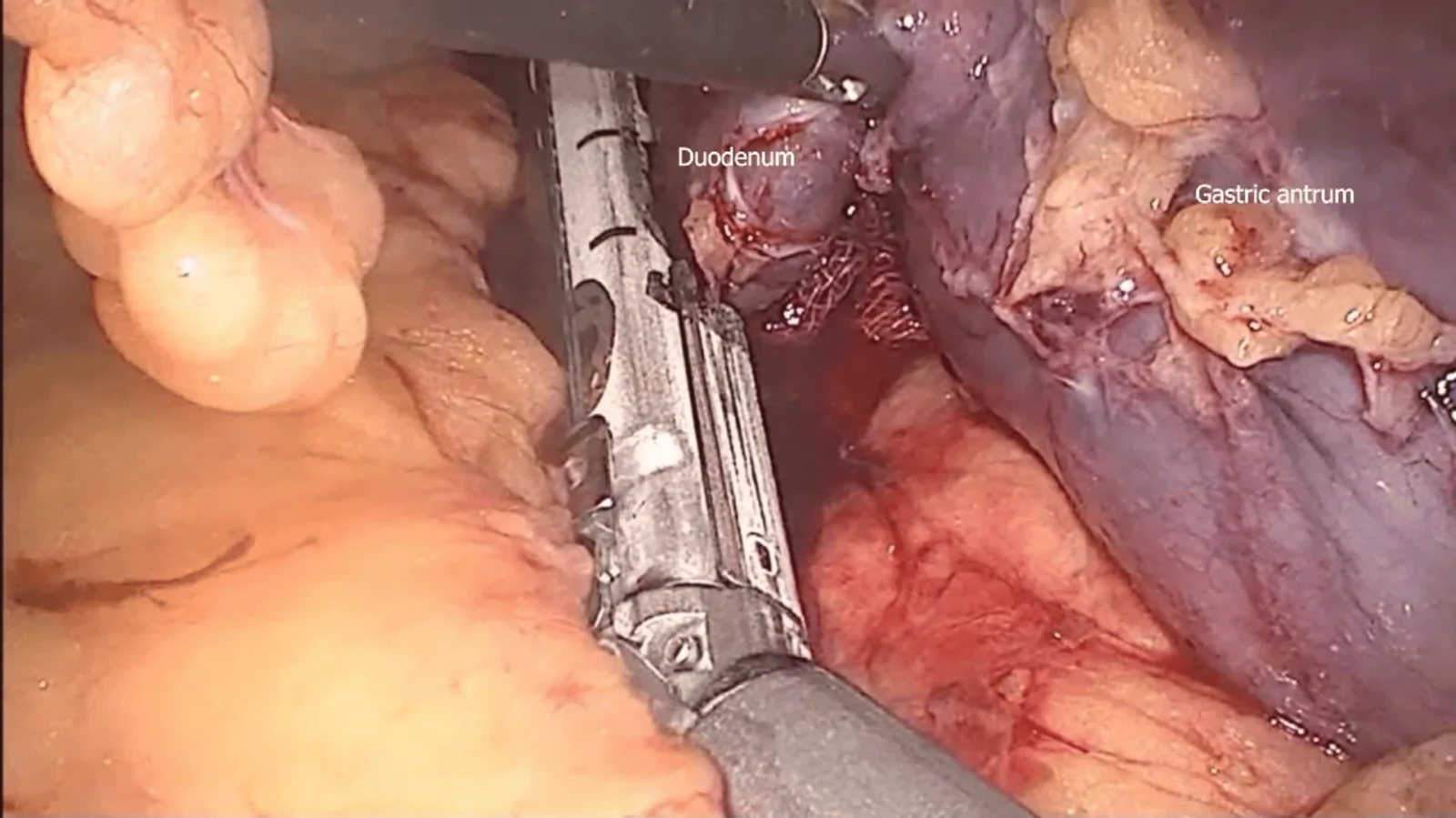
Duodenal transection. Following mobilization of the greater gastric curvature, the first portion of the duodenum was transected using an intermediate-thickness triple-row linear stapler to prevent distal progression of the caustic injury.

The gastrocolic ligament is divided, gaining access to the lesser sac. Dissection proceeds toward the duodenum, and its first portion is transected in order to prevent further passage of caustic below the pylorus (Figure 2). The duodenal stump may be reinforced with a continuous oversewing suture to reduce the risk of fistula and dehiscence. If the corrosive agent has already passed beyond the pylorus, gentle irrigation with normal saline may be performed through the nasogastric tube to enhance dilution of the caustic substance and potentially mitigate its downstream toxic effects on the intestinal mucosa. Then, the gastric fundus is fully mobilized by dividing the left gastroepiploic artery and short gastric vessels.

3. Mobilization of the Lesser Curvature

**Figure 3:**
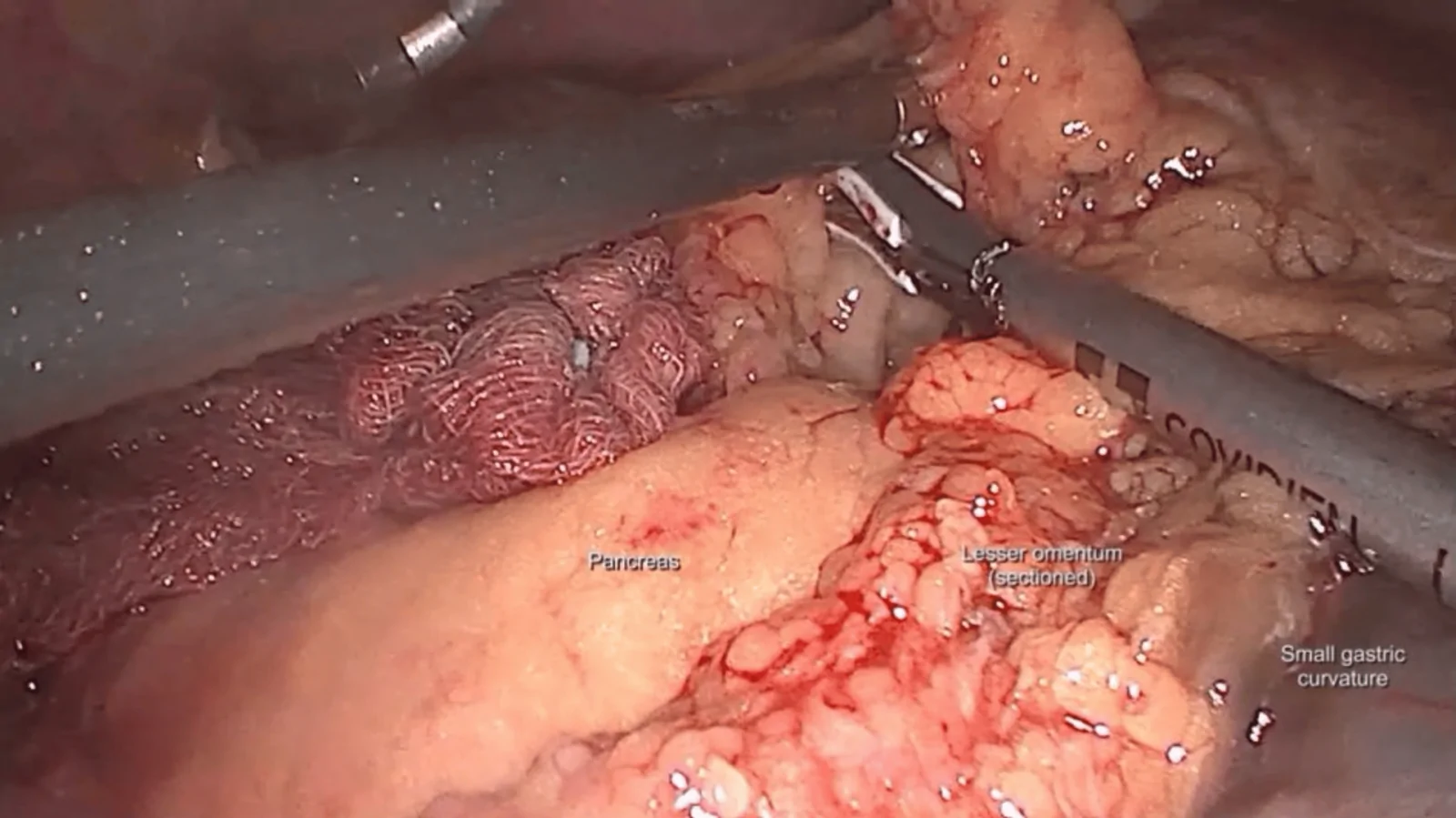
Lesser sac. Division of the lesser omentum (sectioned) and caudal traction of the lesser gastric curvature provide access to the lesser sac.

Division of the lesser omentum exposes the right diaphragmatic crus. Lesser curvature is mobilized, allowing the exposure and division of the left gastric vessels (Figure 3).

4. Transhiatal Mediastinal Dissection

**Figure 4:**
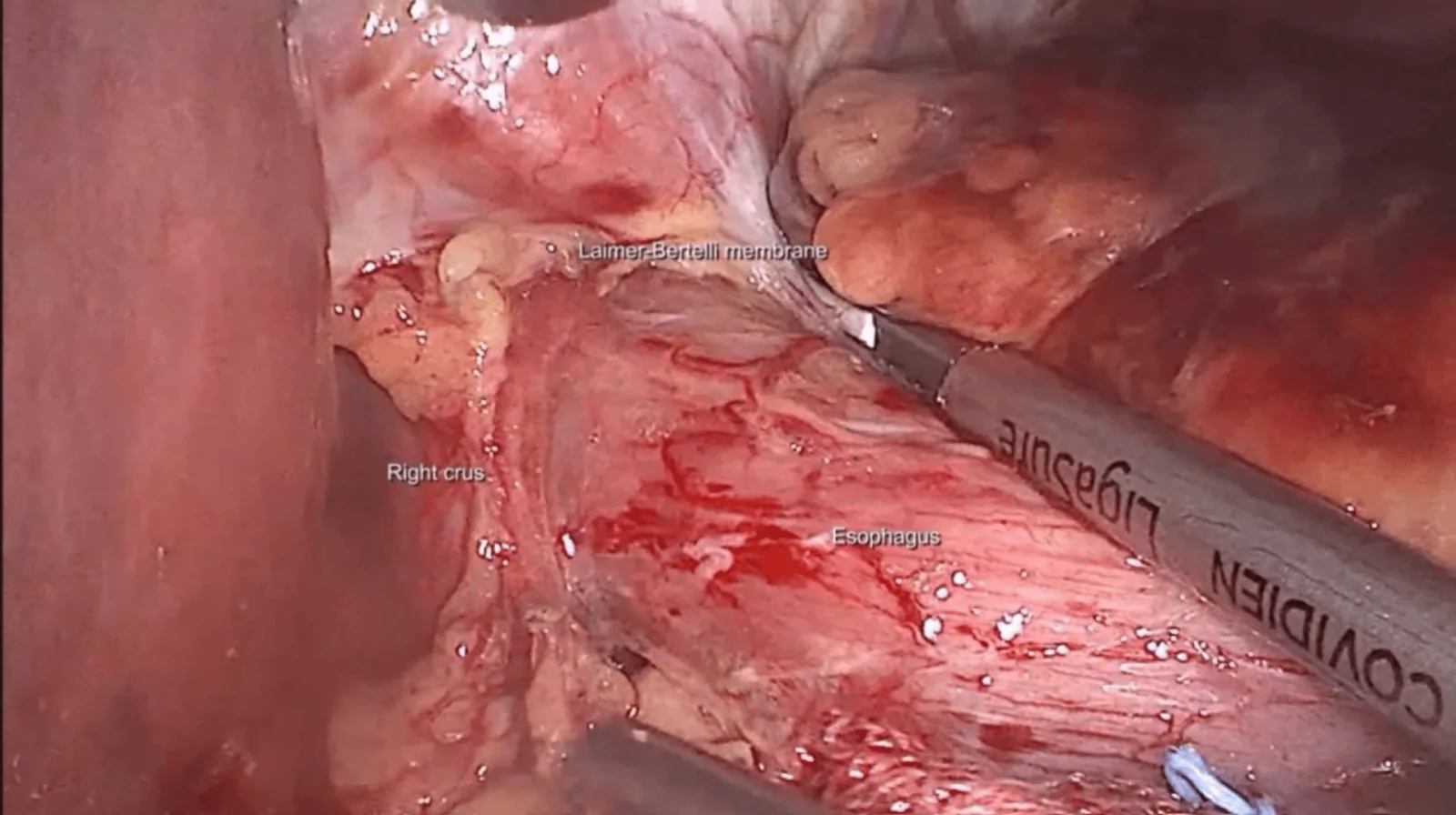
Esophageal hiatus. Circumferential mobilization of the esophagus at the hiatus allows access to the lower mediastinum and the initiation of transhiatal dissection.

**Figure 5:**
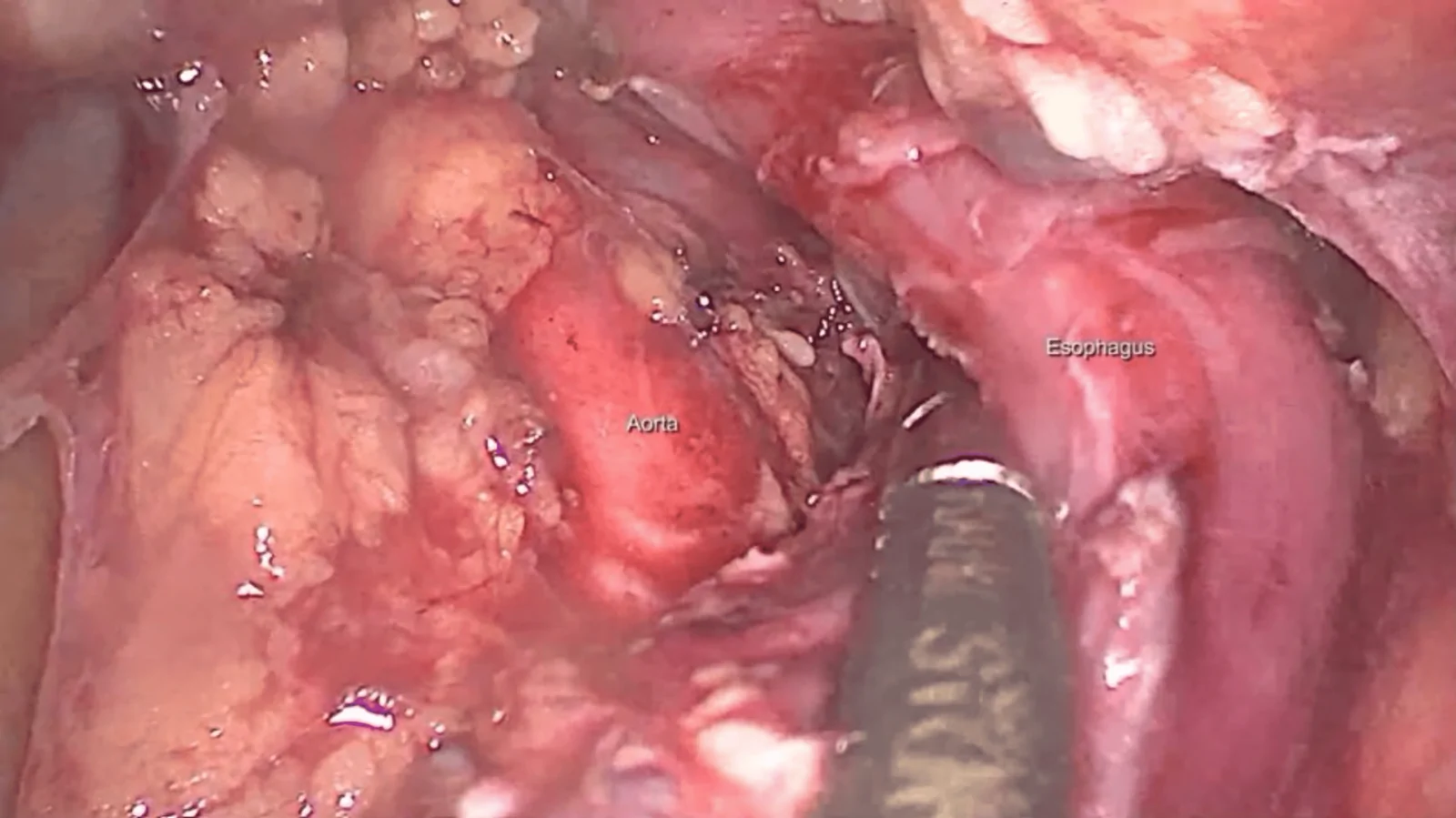
Transhiatal mediastinal dissection. Lateral retraction of the mediastinal esophagus with posterior dissection along the aortic plane.

Dissection of the cardia from the diaphragmatic crura allows access to the inferior mediastinum (Figure 4). Transhiatal mediastinal dissection of the esophagus proceeds along the aortic plane (Figure 5). The superior limit is the pericardium, and the lateral boundaries are the mediastinal pleura. Dissection proceeds in close proximity to the esophageal wall up to the superior thoracic inlet. Complete mediastinal tunneling will be finalized via the cervical approach by opening the endothoracic fascia.

5. Cervical Esophagostomy

**Figure 6:**
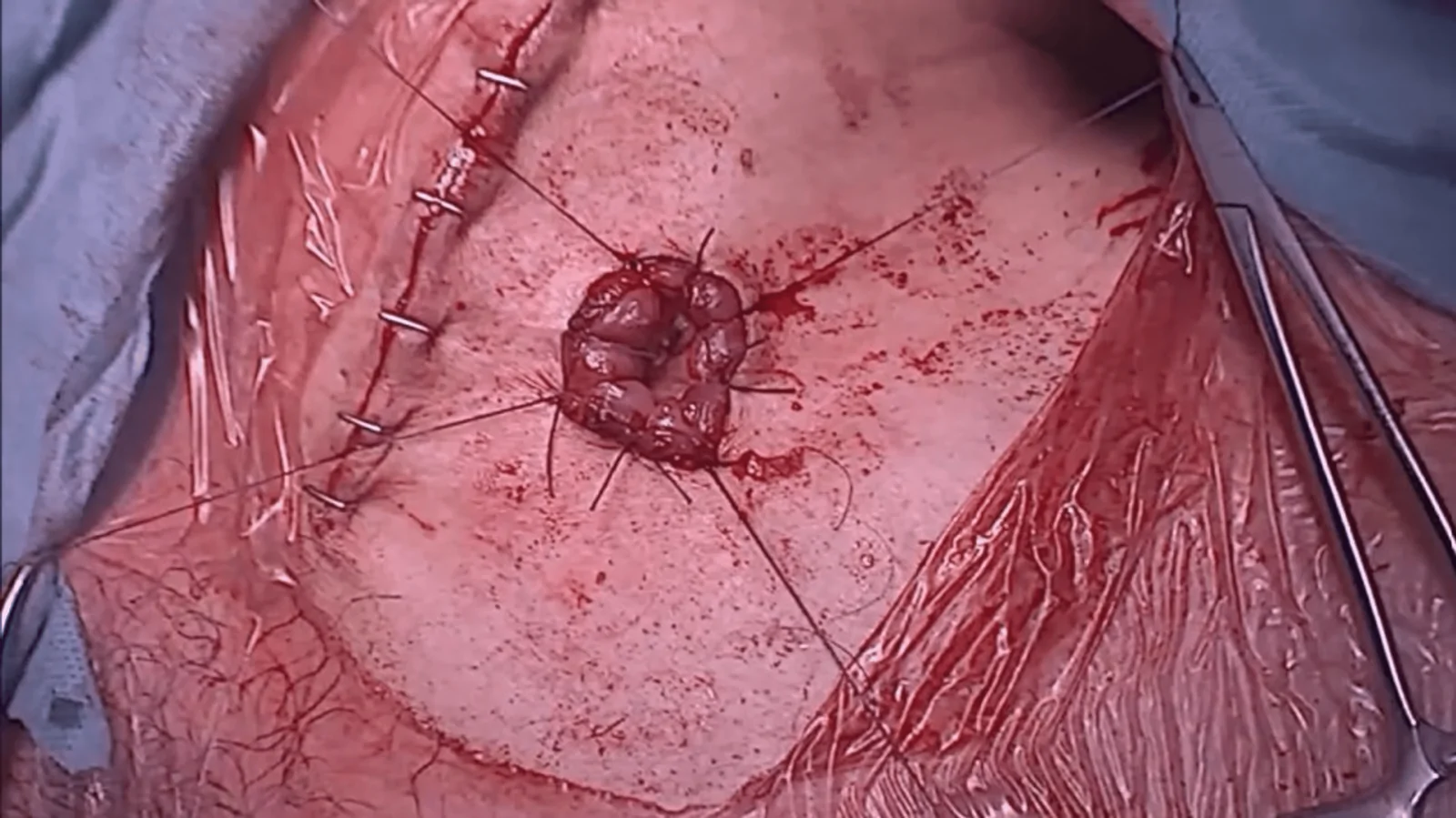
Cervical esophagostomy. A left lateral cervical end esophagostomy was fashioned using interrupted full-thickness 3/0 sutures.

The cervical phase involves a skin incision along the anterior border of the left sternocleidomastoid muscle. After incising the superficial cervical fascia, the platysma is divided, exposing the middle cervical fascia. Sternocleidomastoid muscle is retracted laterally, while the sternohyoid and sternothyroid muscles are retracted medially. Caution is needed due to close proximity to the neurovascular bundle, left recurrent laryngeal nerve, and thoracic duct, which may run near the left jugulosubclavian junction. The esophagus is looped, mobilized from the thyroid, transected, and exteriorized through a passage posterior to the sternocleidomastoid muscle. For adequate fixation of the terminal esophagostomy, each full-thickness suture should incorporate both muscular and mucosal layers (Figure 6). The level of esophageal transection is determined by the location and extent of irreversible esophageal mucosal injury. As a general principle, every effort should be made to preserve the maximum possible length of viable esophagus. At the cervical level, transection should be performed as distally as feasible, close to the superior thoracic inlet, in order to ensure sufficient esophageal length for the safe construction of a cervical esophagostomy without excessive tension. This strategy also facilitates the subsequent reconstructive stage, as additional esophageal tissue may need to be resected to achieve a well-vascularized, tension-free esophagocolic anastomosis.

6. Feeding Jejunostomy

**Figure 7:**
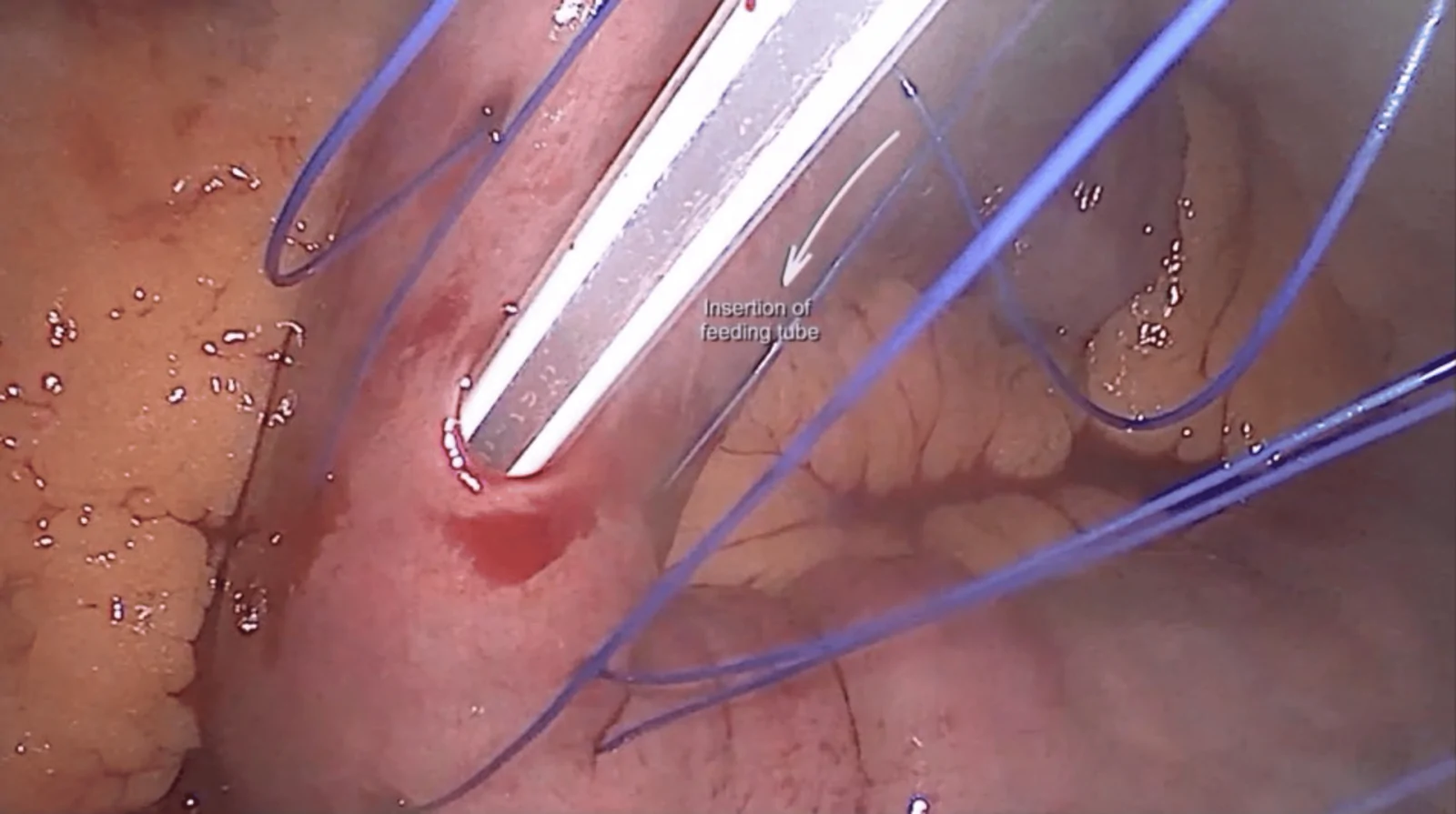
Feeding jejunostomy. Identification of a jejunal loop 25 cm distal to the ligament of Treitz and creation of a feeding jejunostomy, secured to the left abdominal wall with four cardinal slowly absorbable sutures.

A proximal jejunal loop approximately 25 cm distal to the ligament of Treitz is identified. Four 3-0 slowly absorbable cardinal stay sutures are placed percutaneously and secured to the anterior abdominal wall. A small enterotomy is then created to expose the jejunal mucosa. A guide tube is introduced, over which a dilator is advanced. After removal of the dilator while maintaining the guide tube in position, the definitive feeding tube is advanced over the guide. Under direct laparoscopic vision, the feeding tube is gently advanced approximately 20 cm distally within the jejunal lumen. The tube is then flushed with normal saline to confirm luminal patency and exclude kinking or twisting. Finally, the four cardinal stay sutures are tightened externally, completing fixation of the feeding jejunostomy (Figure 7).

The esophagus is delivered into the abdominal cavity, and the surgical specimen is extracted through a small supraumbilical laparotomy. 

Reconstructive phase

The reconstructive phase includes a xiphopubic laparotomy, a video-assisted transhiatal mediastinal tunneling and a left lateral cervicotomy. It was performed after a mean interval of 9.5 months (median 9.5, range 8-11), with a mean operative time of 516 minutes (median 510, range 480-565) and a mean hospital stay of 23 days (median 20.5, range 20-33). A contrast swallow study was performed on postoperative day 7, followed by gradual reintroduction of oral intake. Patient characteristics, operative time, major postoperative outcomes, and the length of follow-up are summarized in Table 2. No surgical reoperations or anastomotic leaks occurred. The reconstructive phase is described in the step-by-step sections below and is further illustrated in the accompanying surgical videos (Videos 2-3).

**Table 2:** Operative and postoperative outcomes of reconstruction phase. Clavien-Dindo, Clavien-Dindo classification of surgical complications; POD, postoperative day

Patient	Sex	Age (years)	Operative time (min)	Postoperative complications (Clavien-Dindo)	Length of hospital stay (days)	Length of follow-up (months)	Symptoms
2	M	45	520	Postoperative ileus (Grade I)	20	41	Night-time reflux and halitosis
3	F	54	500	Pleural effusion (Grade I)	20	34	Postprandial nausea
4	M	44	480	Wound infection (Grade I)	21	7	None
5	F	80	565	Transient dysphonia (Grade I), chylothorax (Grade II), wound infection (Grade II)	33	6	Occasional dysphagia and regurgitation

**Video 2:** Esophagocoloplasty. Reconstructive phase (Steps 7-9).

**Video 3:** Esophagocoloplasty. Reconstructive phase (Steps 10-12).

7. Clamping of the Middle Colic Vessels

**Figure 8:**
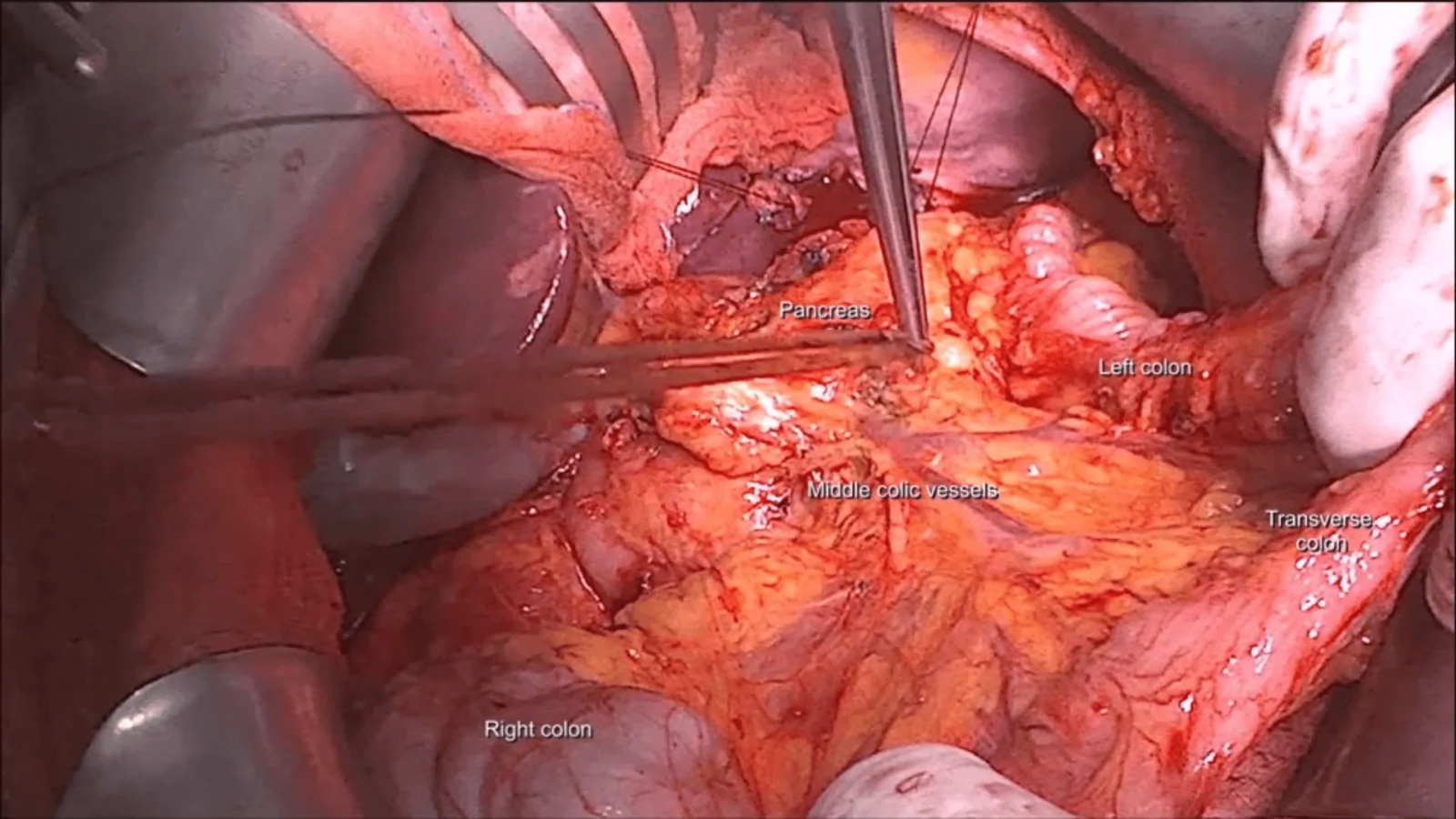
Identification of middle colic vessels. After mobilization of both the right and left colon, ventral traction of the transverse colon allows identification of the transverse mesocolon and the middle colic vessels, which are isolated and clamped at their origin while preserving a patent and communicating bifurcation.

After taking down the feeding jejunostomy, the right and left colon are fully mobilized. Middle colic vessels are separately isolated and clamped at their origin, preserving their bifurcations to ensure perfusion through the marginal arcade from the left colic vessels (Figure 8). Right marginal vessels are also closed. Then, venous outflow from the transverse colon drains via the bifurcation through the marginal arcade into the left colic vein and subsequently into the inferior mesenteric vein. Arterial inflow from the left colic artery follows the same bifurcation through the marginal arcade to perfuse the transverse colon.

8. Cervical Esophagus Isolation

**Figure 9:**
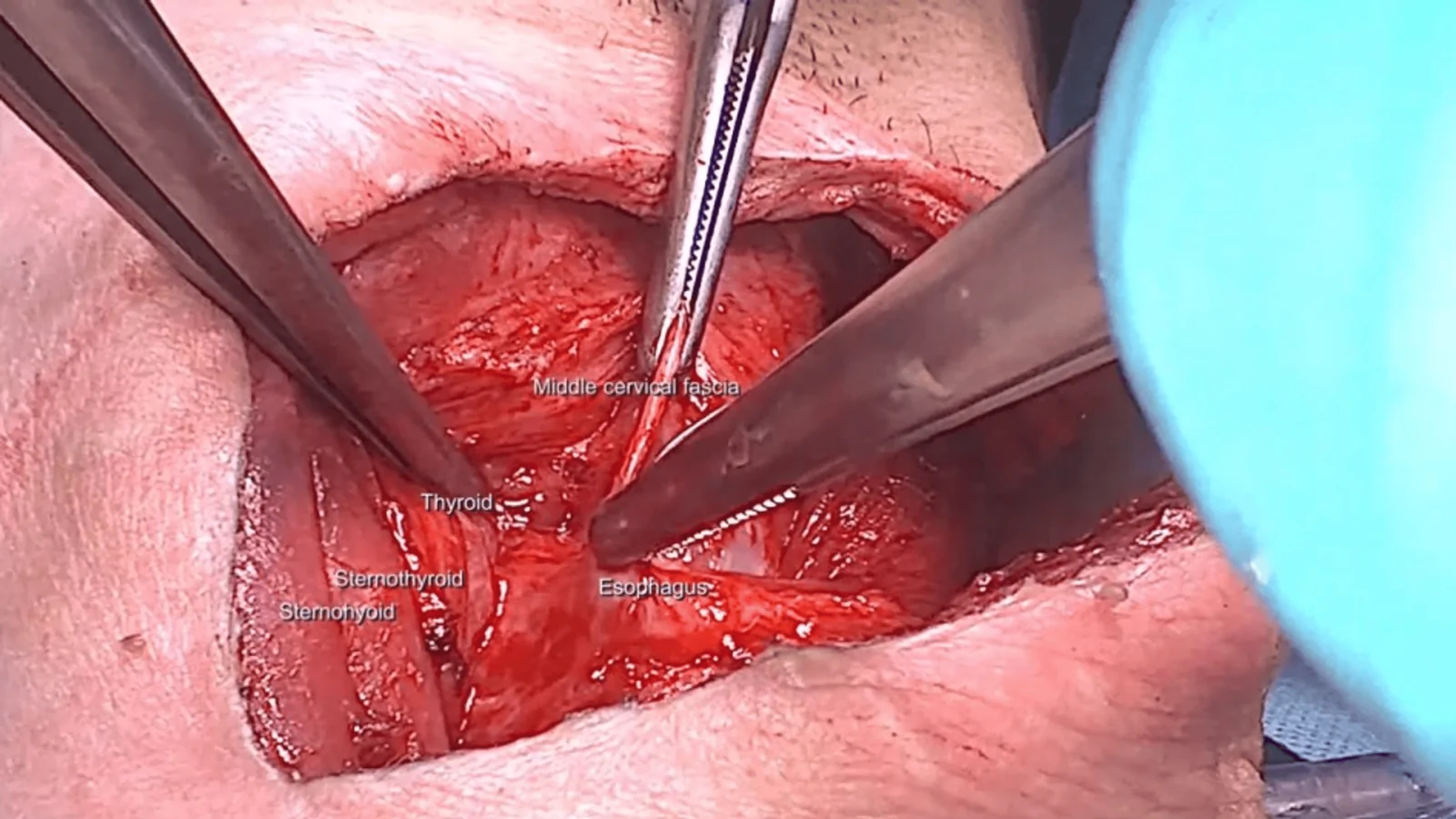
Cervical esophagus isolation. Medial retraction of the prethyroid muscles and the left thyroid lobe allows identification of the esophagus, which lies in a horizontal plane. Esophageal dissection is facilitated by the presence of a nasoesophageal tube used as a landmark.

Cervical dissection starts with a skin incision over the previous surgical scar. Esophagostomy lies horizontally (Figure 9); identification and dissection of cervical esophagus is facilitated by the presence of the nasoesophageal tube. A peristomal skin incision is made, and the esophageal stump is reinternalized into the cervical cavity. Following complete cervical esophageal mobilization, dissection proceeds into the superior thoracic inlet to reach the mediastinum and facilitate abdominal tunneling.

9. Video-Assisted Transhiatal Mediastinal Tunneling

**Figure 10:**
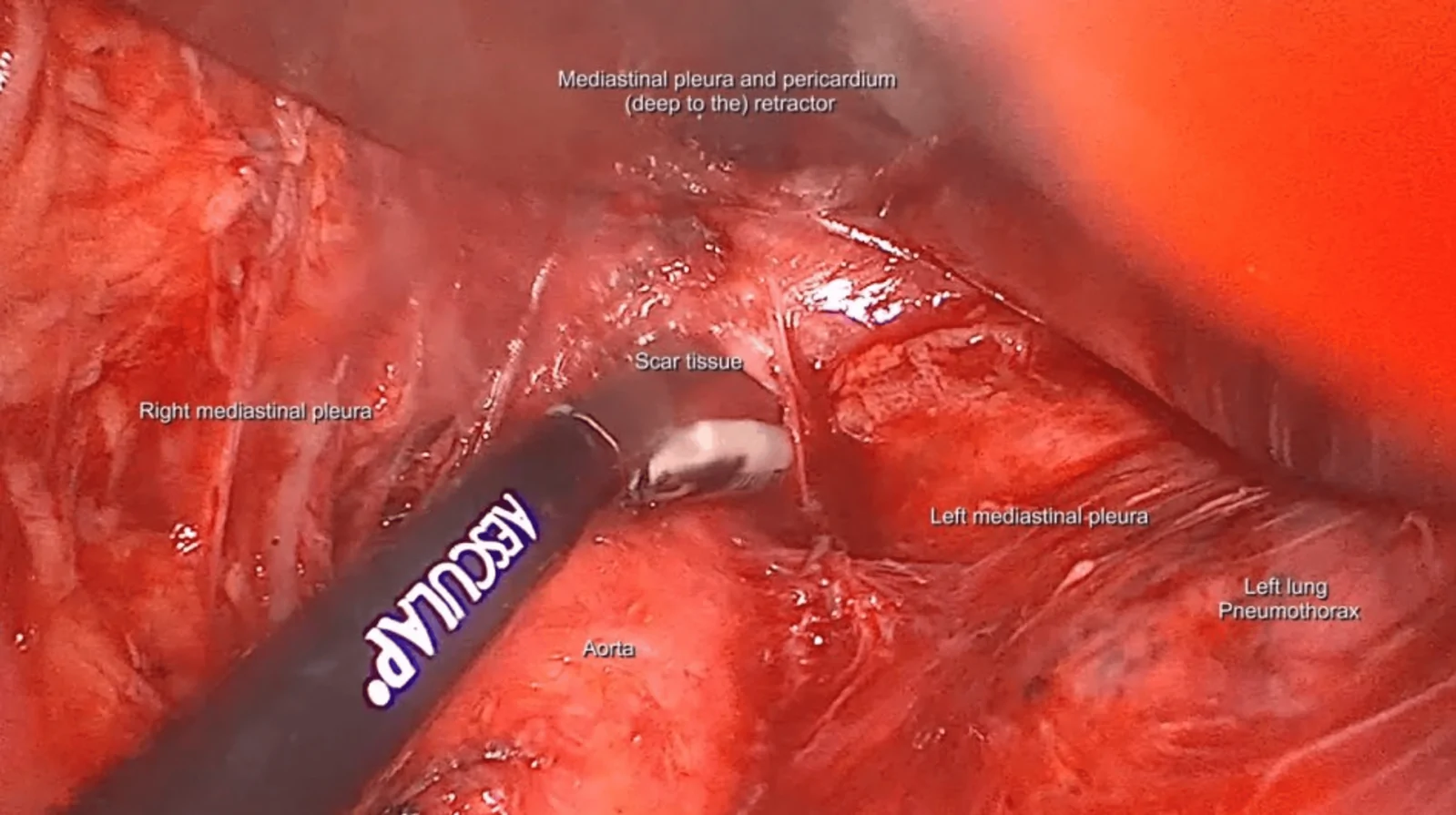
Video-assisted transhiatal mediastinal tunneling. Video-assisted transhiatal mediastinal dissection proceeds in a caudo-cranial direction (from the abdomen toward the cervical region), posteriorly along the aortic plane, laterally along the mediastinal pleura, and anteriorly along the pericardium up to the thoracic inlet. During this step, a left pneumothorax occurred (lower right panel). The intraoperative left-sided pneumothorax was managed at the end of the procedure by placement of a left chest tube connected to suction through the fifth intercostal space, between the anterior and mid-axillary lines.

Diaphragmatic crura are divided, and dissection proceeds through the esophageal hiatus into the mediastinum along the aortic plane using a video-assisted approach (Figure 10). Upon successful transcervical-abdominal connection, an orogastric tube is advanced from the neck into the abdominal cavity to deliver the colonic segment to the cervical field.

10. Assessment of the Colonic Conduit and Transposition

**Figure 11:**
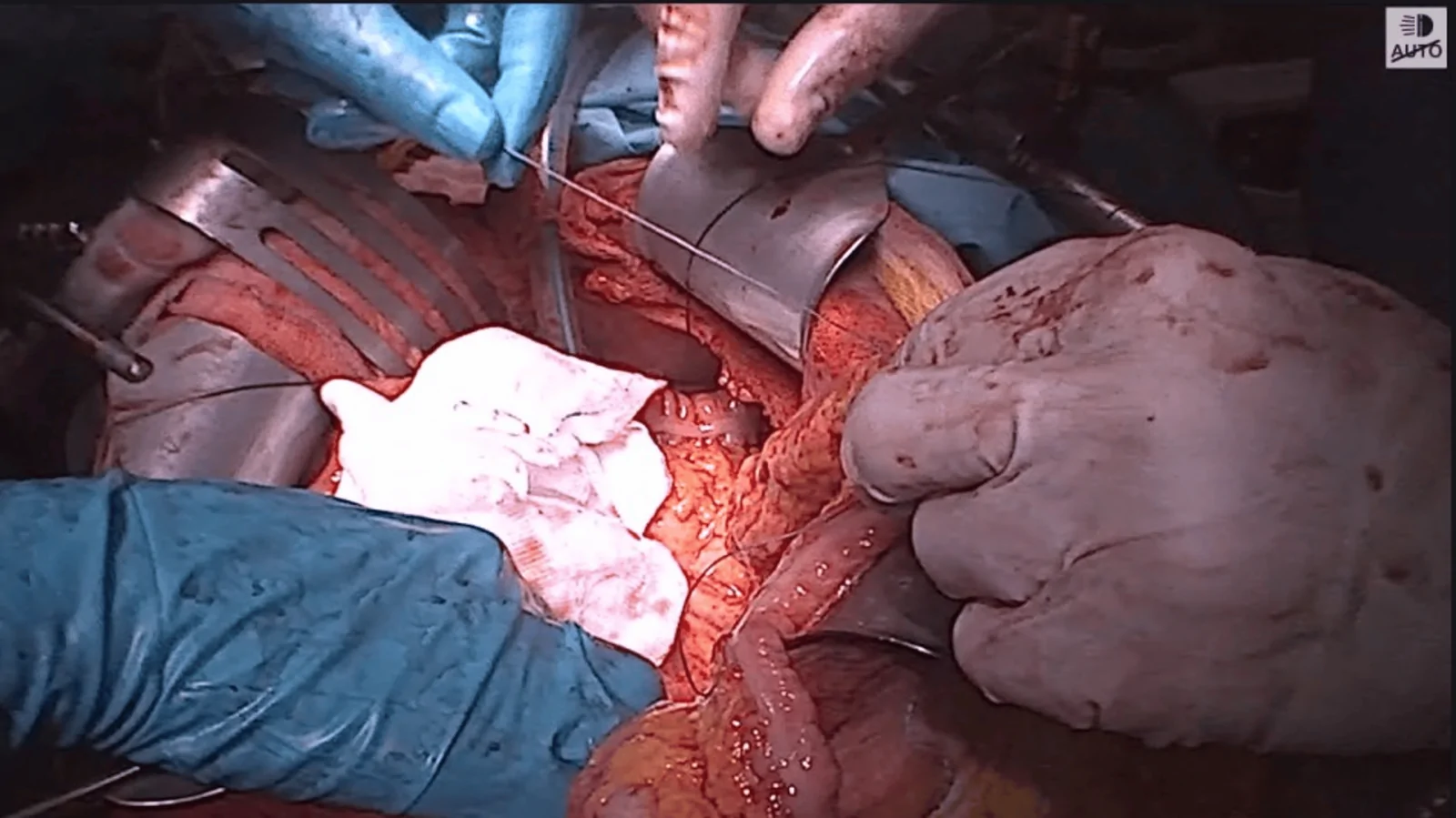
Assessment and measurement of the transverse colonic conduit. A suture is used to measure the distance between the angle of the left mandible and the origin of the inferior mesenteric artery. This measurement is then replicated on the colon starting from the origin of the inferior mesenteric artery. After identifying the segment of descending and transverse colon to be transposed, the sigmoid colon is divided distal to the left colic artery, and the transverse colon is transected at the predetermined measured point.

**Figure 12:**
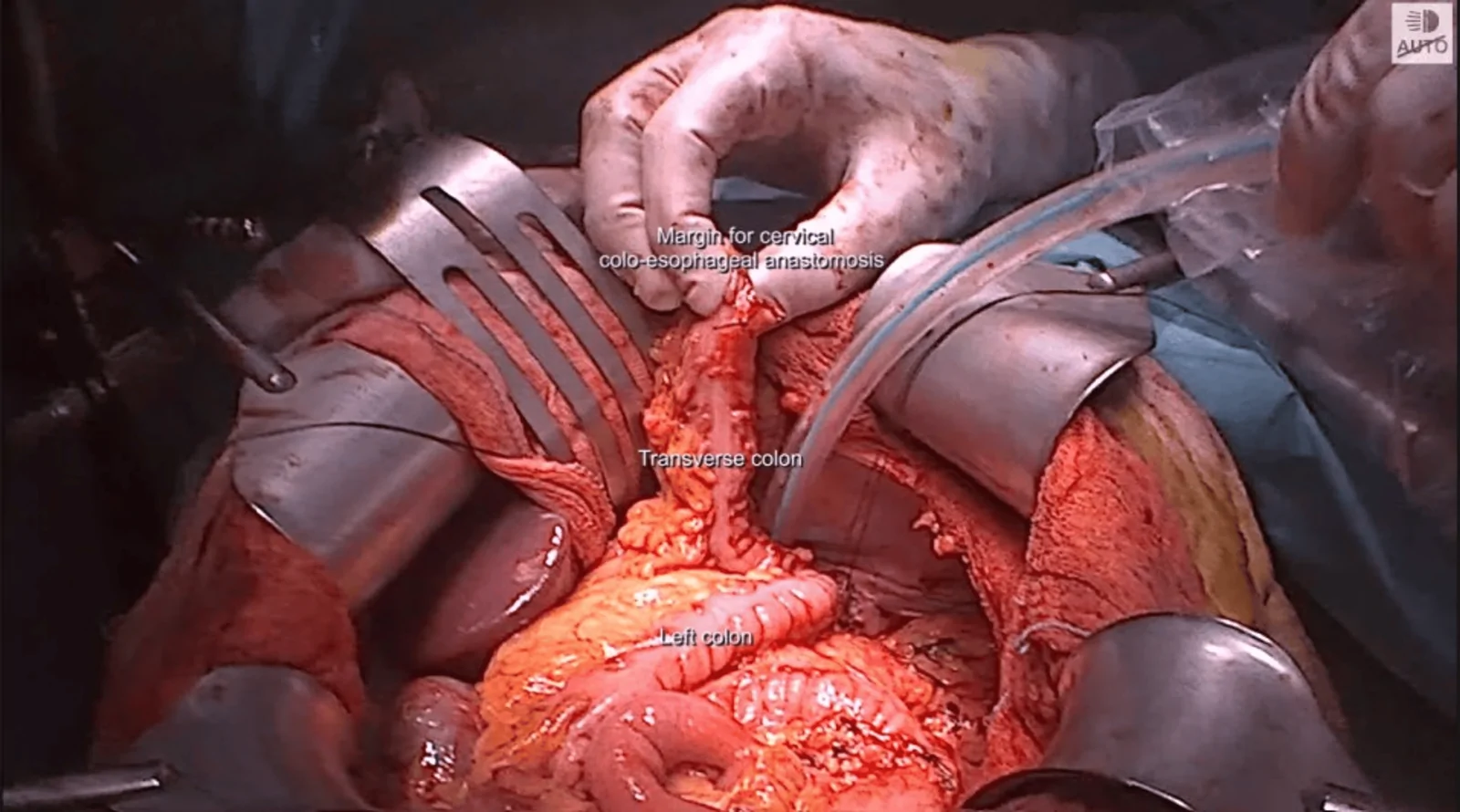
Cervical transposition of colonic conduit. Proximal end of the transverse colon is secured to a trans-mediastinal tube, and the conduit is transposed through the posterior mediastinum using a combined push-pull maneuver between the abdominal and cervical fields.

Following prolonged temporary clamping of the ileocolic, right colic, and middle colic vessels, the adequacy of blood flow through the marginal artery, sustained exclusively by the left colic vessels, is assessed both visually and by palpation. Then, the distance between the angle of the mandible and the origin of the inferior mesenteric artery is measured and replicated along the left and transverse colon to determine the appropriate length of the colonic conduit (Figure 11). The left colic artery must be preserved, ensuring that its vascular pedicle reaches the esophageal hiatus without tension. The middle colic vessels are divided at their origin while preserving the arterial bifurcation to maintain the integrity of the marginal artery and ensure adequate conduit perfusion through the left colic artery. The transverse colon is then transected at the predetermined site. The mesocolon is subsequently dissected free from the retroperitoneum along the inferior border of the pancreas to achieve complete mobilization. The descending colon is transected distal to the left colic artery. At this stage, the colonic conduit is fully mobilized (Figure 12). Its proximal end is secured to a transmediastinal guiding tube, and the conduit is delivered through the posterior mediastinum using a combined push-pull maneuver performed simultaneously from the abdominal and cervical fields. Throughout this step, meticulous care is taken to avoid torsion, kinking, or injury to the bowel, mesentery, and vascular pedicle.

11. Esophagocolic Anastomosis

**Figure 13:**
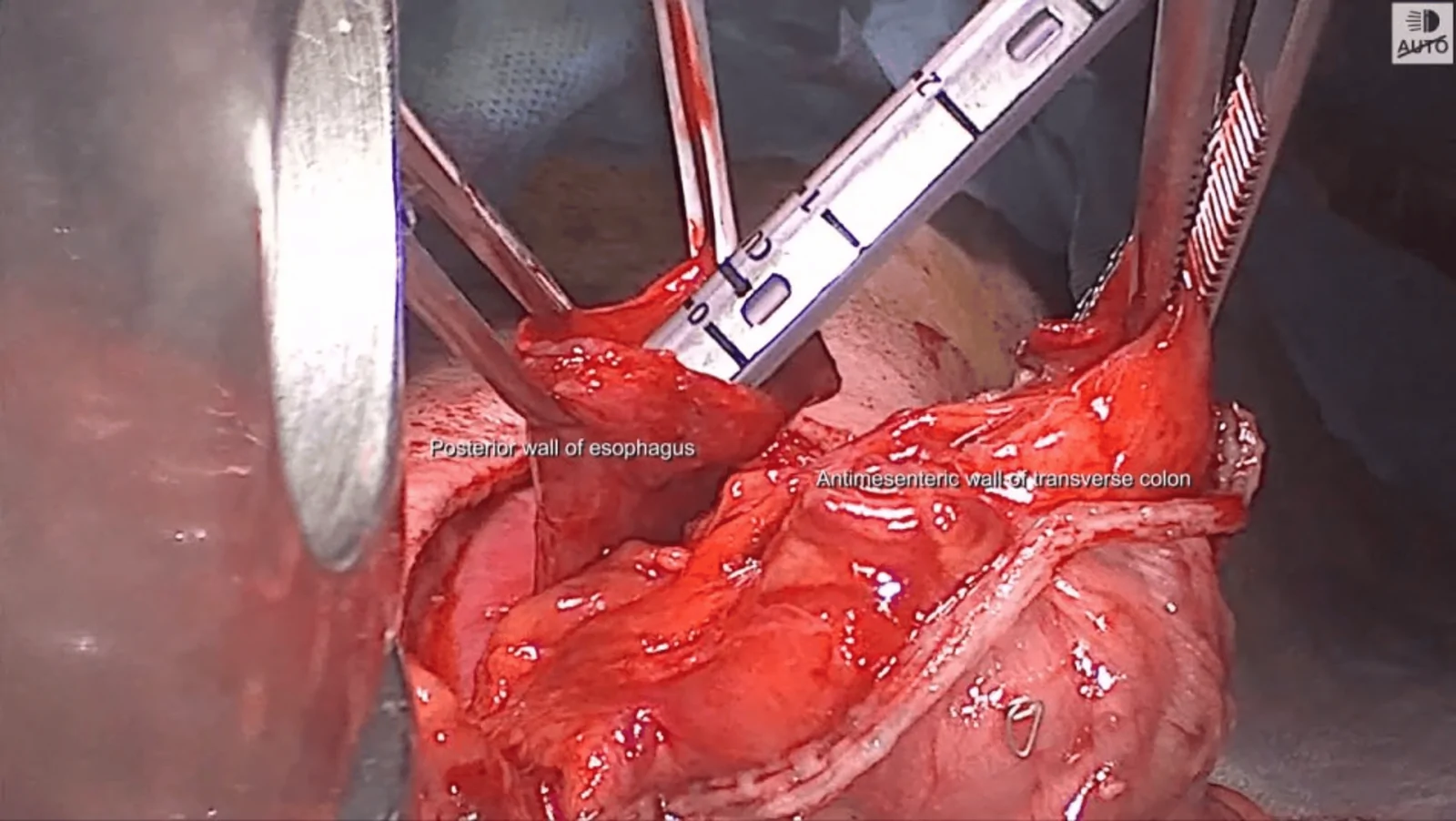
Esophagocolic anastomosis. A mechanical side-to-side cervical esophagocolic anastomosis was performed using a linear stapler (intermediate cartridge, triple-row staple line), with closure of the enterotomy defect in a single-layer fashion using two semi-continuous 3-0 monofilament sutures.

A side-to-side isoperistaltic mechanical anastomosis is performed, and the enterotomy is closed with two hemi-continuous sutures in a single-layer fashion (Figure 13). The skin-matured end of the cervical esophagostomy and the stapled edge of the colonic conduit subjected to traction are resected to eliminate potentially compromised tissue and optimize anastomotic perfusion. The posterior wall of the esophageal stump and the antimesenteric border of the colonic conduit are aligned and anastomosed using a 60-mm triple-row linear stapler with a medium-thickness cartridge. A transanastomotic nasocolonic tube is then advanced under direct vision. The common enterotomy is closed with two running hemi-sutures using 3-0 monofilament. An intraoperative methylene blue leak test is subsequently performed to confirm the integrity of the anastomosis. Finally, a perianastomotic Penrose drain is positioned in the cervical region and brought out through the previous esophagostomy site.

12. Colojejunal and Colocolic Anastomosis

**Figure 14:**
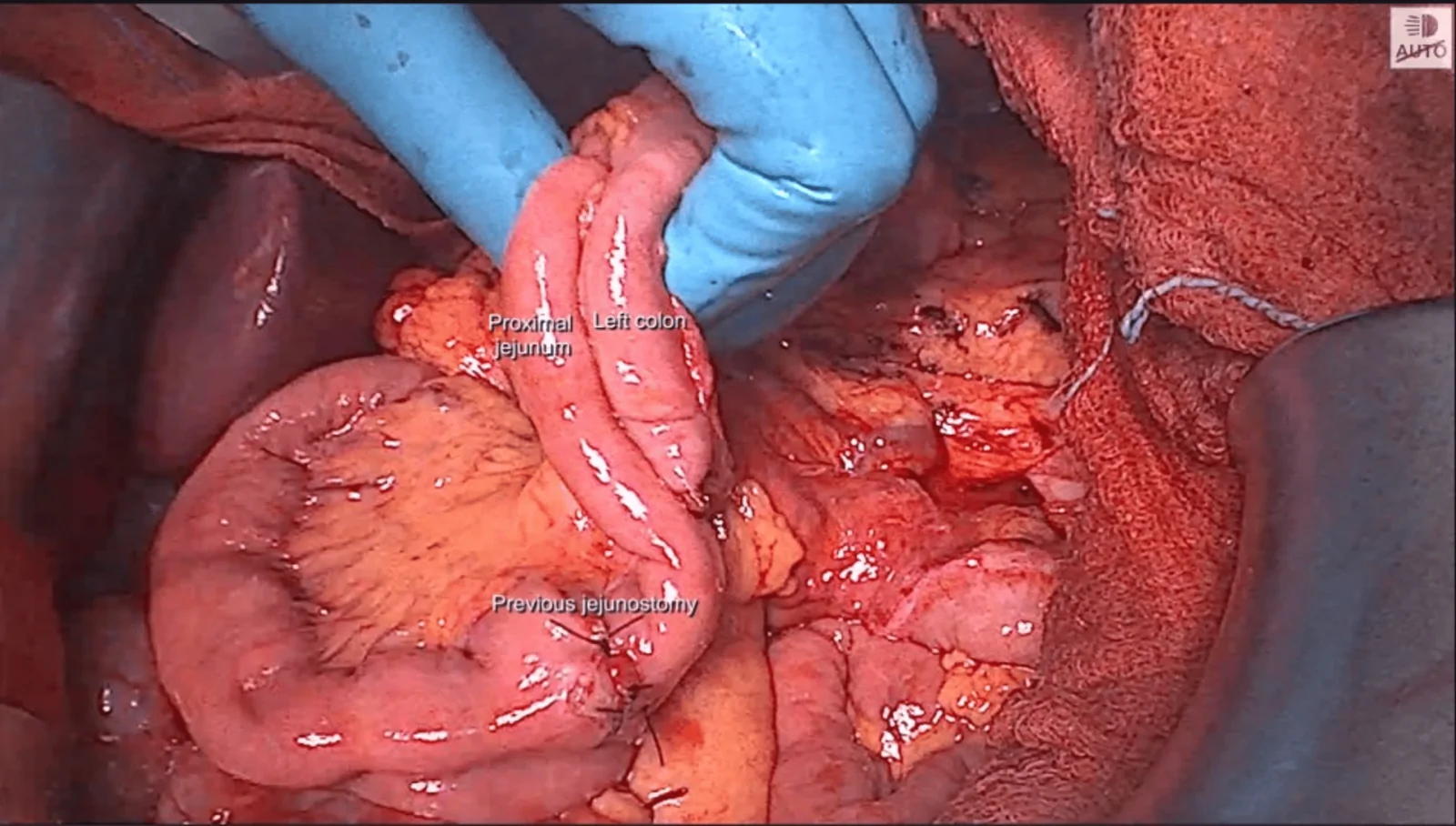
Colojejunal anastomosis. An isoperistaltic side-to-side colojejunostomy was performed using a linear stapler (intermediate cartridge with triple-row staple line), with single-layer closure of the enterotomy defect using a 3-0 absorbable suture.

**Figure 15:**
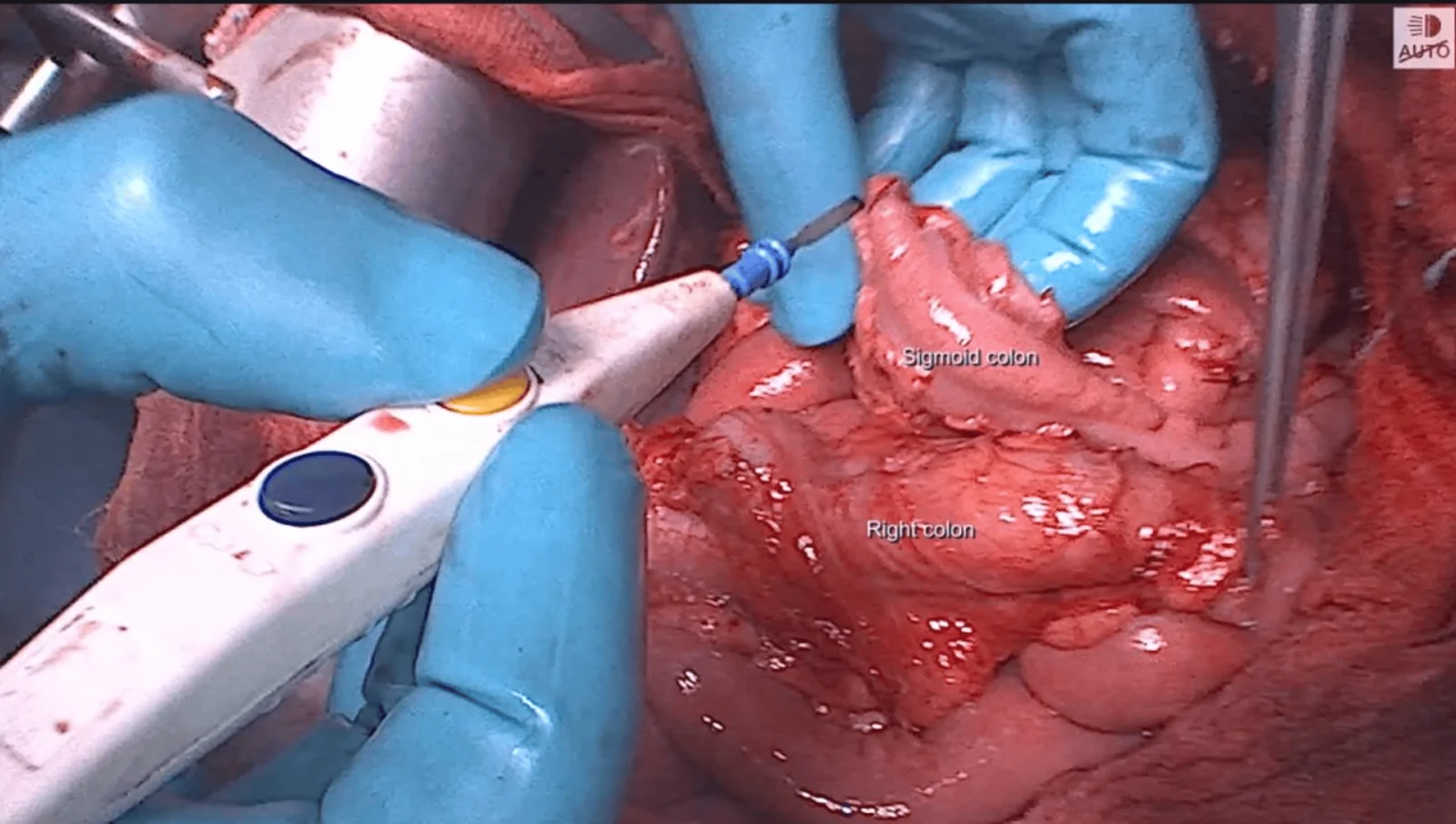
Colocolic anastomosis. An isoperistaltic side-to-side colocolic anastomosis was constructed using a linear stapler (intermediate cartridge, three-row staple line), with single-layer closure of the enterotomy defect using a 3-0 suture.

**Figure 16:**
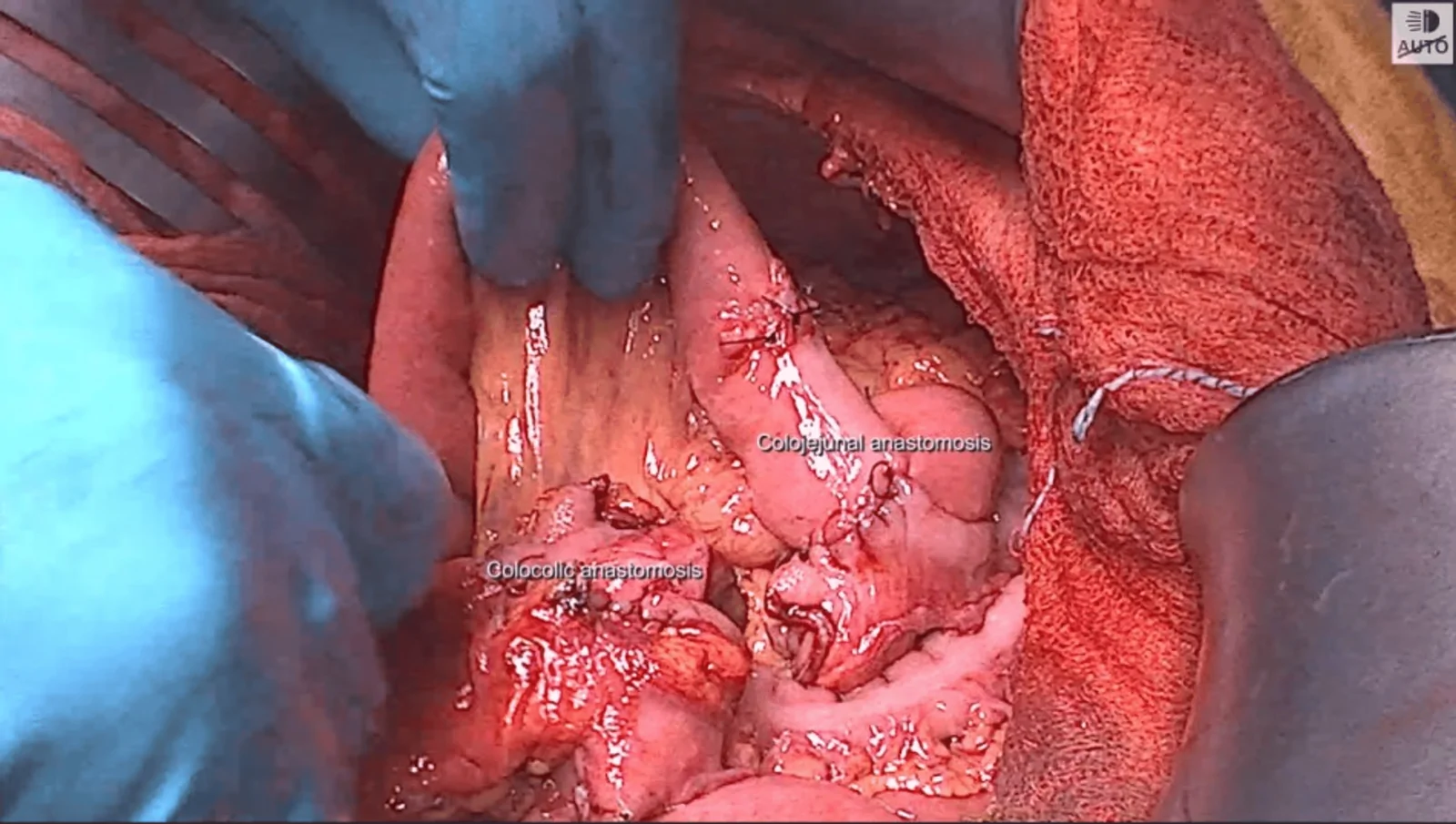
Final anatomical reconstruction. Colojejunal and colocolic anastomosis completed the reconstruction, with restoration of alimentary continuity.

The distal stump of the colonic conduit is anastomosed to the jejunum using a side-to-side isoperistaltic mechanical technique (Figure 14). The distal end of the colonic conduit and the proximal jejunum are aligned. Enterotomies are created on both segments, and a 60-mm triple-row linear stapler with an intermediate cartridge is introduced to fashion a side-to-side colojejunal anastomosis. The common enterotomy is closed in a single layer using a 3-0 absorbable suture. Anastomotic integrity and patency may be assessed manually by gently milking the colonic contents across the anastomosis into the jejunum. Likewise, the right colonic stump is anastomosed to the sigmoid colon (Figure 15), thereby restoring intestinal continuity (Figure 16). The proximal right colon and the distal sigmoid colon stump are aligned. Colotomies are created on both segments, and a 60-mm triple-row linear stapler with a medium-thickness cartridge is introduced to fashion a side-to-side colocolic anastomosis. The common colotomy is then closed in a single layer using a 3-0 absorbable suture. Anastomotic integrity and patency may be assessed manually by gently milking the contents of the right colon across the anastomosis. Then, a new feeding jejunostomy is created distal to the colon-jejunal anastomosis.

## Discussion

Caustic ingestion is an uncommon but potentially life-threatening clinical condition with high morbidity and mortality. Given the rarity, the limited personal experience, and the heterogeneity of surgical strategies described in the literature, a standardized and reproducible approach aimed at improving outcomes is needed [1,3].

Among the surgical strategies adopted for severe caustic injuries, total esophagogastrectomy remains one of the most commonly performed procedures [1]. At present, open surgery combining laparotomy and lateral thoracotomy or transhiatal blunt esophageal stripping through a closed chest remains the standard surgical approach for esophagogastric emergencies [5-8]. In this challenging setting, several authors have already reported the preliminary feasibility of a minimally invasive transhiatal approach, which may potentially reduce the overall burden of staged treatment [6-11].

To the best of our knowledge, only a few reports have addressed the surgical management of this rare and challenging condition [7-9], primarily providing concise procedural descriptions rather than a full report of a standardized surgical technique. Furthermore, none has been accompanied by a comprehensive operative full-length video in a stepwise manner, covering both the demolitive and reconstructive phases of the procedure, with particular emphasis on critical technical aspects, key surgical steps, and relevant anatomical landmarks. Such standardization is especially valuable in this uncommon and technically demanding clinical setting, as it may enhance procedural reproducibility, facilitate surgical training, and promote the broader adoption of minimally invasive strategies. Moreover, given that open surgery is still considered the gold standard for the emergency management of caustic ingestion [1,4,5,10], evidence supporting minimally invasive approaches is limited to a small number of surgical reports [7,8,11]. In this context, due to the complexity of the procedure, the limited evidence available in the literature, and the well-established benefits of minimally invasive surgery, a technical step-by-step video report of minimally invasive transhiatal esophagogastrectomy based on damage-control principles, followed by delayed esophagocoloplasty with video-assisted mediastinal dissection, is described in a standardized, educational, and reproducible manner.

Analyzing the surgical technique, the decision to perform a minimally invasive esophagogastrectomy was based on intraoperative findings of irreversible injuries confined to the esophagus and stomach, with no evidence of damage to other organs or viscera. Conversion to laparotomy is recommended in the presence of extensive multivisceral necrosis, diffuse peritonitis, massive hemoperitoneum, and hemodynamic instability. Given the risk of progressive caustic injury, damage control principles are mandatory, prioritizing source control and patient stabilization. In this context, the attempt of single-stage reconstruction has to be avoided [1,4,5].

Opening the esophageal hiatus and dividing the diaphragmatic crura may result in thoracic duct injury and pneumothorax. Owing to the confined mediastinal space, transhiatal mediastinal dissection is technically demanding and carries a risk of major vascular injury involving the aorta, internal mammary vessels, and bronchial arteries, as well as unrecognized tracheobronchial injury. In addition, excessively caudal placement of the abdominal trocars relative to the xiphoid process, together with the limited length of standard laparoscopic instruments, may hinder mediastinal tunneling and adequate exposure of the superior thoracic inlet. To overcome these technical challenges, a more cranial trocar placement and the use of the longer laparoscopic instruments commonly employed in bariatric surgery may facilitate dissection, particularly in patients with a large thoracic cavity.

During the left cervical phase, meticulous attention should be paid to the preservation of critical anatomical structures, including the left recurrent laryngeal nerve, which courses within the tracheoesophageal groove, the parathyroid glands located in close proximity to the inferior thyroid artery, and the thoracic duct, which drains into the left jugulosubclavian venous confluence.

The transcervical re-entry into the superior mediastinum through the opening of Sibson’s endothoracic fascia may occasionally result in apparently significant cervical bleeding, with blood tracking into the abdominal cavity. This complication is usually related to injury of the thyroid ima artery, and prompt ligation of the vessel effectively controls the hemorrhage without compromising thyroid function.

The reconstructive phase was performed after adequate recovery, ensuring resolution of inflammation and optimization of nutritional and psychological status. Preoperative colonic evaluation included a complete colonoscopy to rule out colorectal polyps and diverticular disease, along with CT angiography to assess the colonic vascular anatomy and integrity of the left colonic pedicle. 

The transverse colon was selected due to its reliable vascularization via the left colic artery, favorable diameter, and length. The posterior mediastinal route was preferred to maintain physiological alignment of the alimentary tract, despite potential technical challenges due to adhesions of previous surgery [12].

The emergency laparoscopic approach resulted in minimal intra-abdominal adhesions during the reconstructive phase, with both the small bowel and colon remaining freely mobile. The only constant finding was adhesion of the residual omentum to the esophageal hiatus, which was found to be obliterated, likely due to cyclic mediastinal pressure changes related to respiratory excursions.

Adequate colonic mobilization is crucial to prevent tension on the esophagocolonic, colojejunal, and colocolic anastomosis. The right colon is mobilized in a lateral-to-medial direction along the plane between Toldt’s and Gerota’s fasciae until the caval plane is reached. The left colon is similarly mobilized to the aortic plane, taking particular care to preserve the left colic vessels, which constitute the vascular pedicle of the colonic conduit. Complete mobilization of the transverse colon requires detachment of the transverse mesocolon at the root from the retroperitoneum and the inferior pancreatic border. Following temporary clamping of the middle colic, right colic, and ileocolic vessels, careful visual and tactile assessment of the marginal artery is mandatory to confirm adequate perfusion of the colonic conduit. The marginal arcade is entirely dependent on the left colic artery in this setting, while venous drainage must be verified through the inferior mesenteric vein. Once adequate vascularization of the conduit via the left colic artery and the marginal arcade has been verified, the middle colic vessels may be divided at their origin, preserving their right and left branch bifurcation. 

Posterior transhiatal mediastinal re-tunneling represents one of the most challenging steps of the procedure, as mediastinal fibrosis secondary to previous caustic injury and mediastinitis may lead to obliteration of the esophageal plane, increasing the risk of intraoperative complications. In this scenario, video-assisted mediastinal dissection provided enhanced visualization and facilitated safe identification of the anatomical planes, allowing successful restoration of the native alimentary route without major vascular injuries. A pneumothorax was the only intraoperative complication encountered and did not result in any hemodynamic or respiratory impairment. Consistent with the literature, video assistance was particularly valuable in safely recreating the mediastinal tunnel [7].

During the cervical reconstructive phase, insertion of a nasoesophageal tube facilitated the dissection of the cervical esophagostomy, providing better control of the proximal esophageal stump.

Successful cervical transposition of the colonic conduit requires that the origin of the left colic artery reaches the esophageal hiatus without tension. The conduit must be carefully oriented to avoid twisting or excessive traction, while avoiding excessive length to prevent intrathoracic redundancy and potential functional impairment.

This study has several limitations. First, the rarity of this condition resulted in a limited sample size. Second, the monocentric design may restrict the external validity and generalizability of the results. Third, all procedures were performed by a surgeon with extensive experience in advanced laparoscopy and upper gastrointestinal surgery; therefore, the applicability and reproducibility of this technique in centers with limited laparoscopic expertise remain to be established.

## Conclusions

A minimally invasive transhiatal approach to emergency esophagogastrectomy appears to be technically feasible in carefully selected patients with severe caustic injuries when performed in experienced centers with advanced minimally invasive expertise. This technical report provides a stepwise description of the procedure and supports its preliminary feasibility and potential role within a staged treatment strategy. Given the limited number of patients and the descriptive nature of this series, further studies are required to establish the safety, reproducibility, broader applicability, and standardization of this approach. A staged management strategy based on damage control principles remains a key component in the management of these complex patients.
